# Mr. Hill, on Contraction of the Lower Limbs

**Published:** 1806-04

**Authors:** Geo. N. Hill

**Affiliations:** Chester


### *To the Editors of the Medical and Phyſical Journal.*

"Truths built on observation and experience seldom stand single, they generally lead to others, and become the means of more diffusive knowledge." *Pott, on Palsy of the Lower Limbs, p.* 432.

GENTLEMEN,

RETROSPECTIVE views are sometimes not less useful in the practice of medicine and surgery, than in the political concerns of a well regulated government, where the advancing welfare of the people forms the most prominent feature of the laws and constitution.

That the avidity for something new should increase with advancing knowledge is perfectly natural; science is happily progressive, and whatever of folly, of luxury, or of the evils of misguided ambition, may have designated the close of the last century and the commencement of the present, the most carping cynical admirer of the days that are past, must concede that a Jenner and a Greathead have proved that something has been added to the sum of human consolations.

To confine our view in the present consideration to matters strictly medical, it has long appeared to me no unimportant enquiry occasionally to ask the question, Whether the zeal and ardour for extending and improving the advantages advantages gained by former discoveries of acknowledged reputation keeps pace with the increasing eagerness of pursuit after something new?

Notwithstanding the exertions and experiments of a Withering and a Beddoes, have practitioners yet proved the full virtues of the digitalis? Do we know the full extent of the powers of opium, of arsenic, or of mercury? and are the several combinations of active remedies duly appreciated? Some powerful medicines have been ushered into the world with such exaggerated praise as to lead astray the most cautious, consequent disappointment has prejudiced them against the farther use of agents really possessing virtues of considerable eminence, although not equal to the expectations they had raised; some from their great power, and the caution necessary to their use have been too often laid aside; when it would have been sound policy to have had recourse to their aid. This being the fact, it becomes us (in my poor opinion) sometimes to pause, and to review. When the violent nature of the ambiguous disease which our great predecessor Pott considered as so insiduous in its attack, so slow and yet so sure in its progress, and so destructive in its effects, be reflected upon, that such a mass of mischief should be arrested in its progress, its dreadful effects not only checked, but wholly removed by apparently very inadequate agency; it is impossible not to occasionally consider what must be the power, what the influence of the action excited by the purulent discharge, artificially produced in the neighbourhood of the source of the evil by the use of caustic? A change so great, so universal, and unequivocally capable, singly and unaided by any auxiliary, of producing so mighty an alteration, must undoubtedly deserve frequent contemplation in every case which bears, or brings under our view, any thing like analogy.

In the medical art, it has often been the source of illiberal sneering, that the whim of fashion has not failed to insinuate her capricious and wayward fancies into the conduct of its most enlightened professors. To repel the invidious charge, it may be observed with the strictest regard to truth, that humanity and a constant anxiety to promote the honour and establish the success of the art, have certainly produced much of this avidity for novelty, this extolling of a remedy to-day and its abandonment to-morrow; and, what is more directly applicable to the purpose and intent of this paper, the neglect to which many valuable valuable agents are consigned, not directly perhaps in few instances, but indirectly in many.

That excellent surgeon, my worthy preceptor, Sir W. Blizard, in his Friendly Lectures on a Sunday afternoon, used to observe to his pupils, "After having acquired all the information you can up to your own time, on any subject; after having made yourselves masters of all the knowledge of your predecessors; let it then be your first aim, your grand study, to endeavour to step forward from that spot, and your highest glory to have it in your power one day to say you have advanced, to have made but one successful, one useful progressive movement, by adding to the stock of medical knowledge, will entitle you to rank with the benefactors of the human race."

Having, in the course of the last fifteen years, met with several unhappy cases of severe contraction of the lower limbs, which incapacitated the sufferers from almost every enjoyment of existence, consequently rendering them wretched to themselves and burdensome to those around them: Having also repeatedly witnessed the absolute failure of the whole round of internal medicine, and external applications in producing even partial amendment, much less complete recovery in any one instance, the case now submitted to the Readers of your Journal fell in my way; and a short history of the subject will throw some light on the progress of the mischief.

John Edwards, aged 48, by trade a Mason, never remembers having any illness in his life until the 23d year of his age, when he was afflicted with a severe ague, which lasted nine months, and went off at the height of the summer season spontaneously. At the age of 37, whilst working hard at his trade in the Liverpool Docks, much exposed to damp and wet, he was gradually afflicted with severe pain near the bottom of his back, feverishness, loss of appetite, and cramp; feebleness daily increasing, but the lower limbs suffered but little pain comparatively, and were wholly free from the least degree of contraction; notwithstanding which, he could not assist himself in the smallest degree in turning in bed; and when placed on his feet, although he could stand and walk, yet he could not turn even his head round without turning body and limbs together. He was carried to the infirmary, and remained there several weeks, taking medicine and using the warm bath, friction, &c.; but not gaining ground so fast as he could wish or had expected, he one evening took his departure *sans ceremonie*, returned to his wife and children, took took brimstone, cream of tartar, and as strong a decoction of guaiacum shavings as could be made; in a few weeks the pain and stiffness going off, he returned to his employ, and worked seven years more in good health. His wife dying about the year 1797, he volunteered as a seaman, was appointed to Sir R. Onslow's ship the Monarch, and served under this officer in the action off Camperdown; from thence he went with Admiral Dixson to cruise in the North Seas till the battle of Copenhagen, where he fought in the same ship, then commanded by the brave Captain Moss, under the immortal Nelson; three weeks after this memorable engagement, Edwards was turned over to the Blenheim, where he continued till the peace of Amiens. On being discharged, he came with some fellow sailors in a chaise from Portsmouth to Birmingham; from thence on the top of the stage coach to Liverpool. In this journey he contracted a violent hoarseness, pains in his limbs, and a cough; nevertheless, he resumed his work, and continued it about a fortnight, when pain, feverishness, and weakness, sent him to bed entirely for eight weeks. One night he was suddenly seized with a total immobility of the right leg and left arm; the former was drawn backwards rather more than half way towards the buttock, and wholly immovable, though not very painful, except when attempts were made to straiten it; the arm was similarly affected, excepting that the hand instead of being inclosed inwards, as the arm was at the elbow, was strongly extended outwards, the limb resting high on the thorax. After remaining thus a fortnight he was placed a second time in the infirmary; soon after his admission the other leg and arm assumed a similar state, but were never quite so bad as the first affected; thus he remained wholly helpless for twelve weeks, took a great quantity of medicine and mercury so as to affect his mouth very much, went constantly into the warm bath, and was rubbed with ol. palmæ; his appetite gradually degenerated, emaciation and debility advanced proportionally, but he had now no pain whatever in the vertebral column, nor had he felt any thing of the kind this illness. In this situation, on it being proposed to send him to the poor house, he objected, and was carried on a man's back to a boat, and by that conveyance he arrived among some relatives in this city. A worthy lady in the neighbourhood gave him a recommendatien to our infirmary as an out-patient; he was attended several weeks by Mr. Hughes, house surgeon to the institution, with that attention to his case for which this young practitioner is justly signalized. Tonics of every description, with opium, mercury, and friction, were again tried, with the favorite guaiacum decoction, of which the patient had an high opinion, all with unabated diligence, for several weeks. No adequate improvements appearing, the case was given up, and the sufferer thrown wholly upon his parish. In this hopeless state I first saw him, unable to lie in bed any way but on his back, with the limbs above described, and fed like a child: This was Nov. 10, 1802.

On examining the case, it appeared as completely one of those where scarcely any thing had been left undone that the symptoms indicated could be done, as any I had ever seen. The only idea that held out any feasible prospect of being servicable to the brave British tar, turned upon an accurate examination of the spine; yet to this examination I had no encouragement from any symptom mentioned to me. He retained his urine and fæces perfectly, had no pain in his back, nor any of the peculiar corded stricture affecting the stomach, so accurately noted by Mr. Pott; in short, there existed no solid evidence that he laboured under such disease as that so admirably described by our great predecessor. On causing him to be raised, this state of matters was confirmed; nothing seemed amiss with any part of the spine, from the atlas to the sacrum and coccyx. The urgent intreaties of the poor fellow induced me to try a new course of tonics; he took iron in various shapes, decoction of the woods, alkaline sudorifics, nitrous acid, alternated with the mineral arseniated solution, opium and bitters, the most generous food his stomach would bear, and he could procure, the constant and liberal use of fomentation and friction, with linim. ovi. ol. ped. bov. &c. administered twice a day.— Thus we persevered to the end of Sept. 1803. He had now acquired a little return of appetite and strength, was able to sit upon a chair an hour daily, had somewhat less pain when the limbs were bent; but this was all the result of great exertion to obtain a cure. I had nothing more to propose but country air, time, continuance of friction and fomentation. Thus the winter of 1803 and spring of 1804 were passed. In May, he could sit up half a day; now and then his limbs were somewhat more flexible, the arms better than the legs, though he could not yet bear crutches. He was now admitted an in-patient of the Chester Infirmary, under the care of Dr. Cuming, who gave his case considerable attention. He bathed again, and took took tonic medicines, with a pill, which sweated him at night; but remained, after a trial of six weeks, much the same. He was carried home on the porter's back; and from thence, a few we[illegible]s after, to his parish, (Moldin, in Flintshire) in a ca[illegible]t; after which journey he was fully as great a cripple as at any time during his illness. I did not see him again till May, 1805; he now, by recurrence to the old powerful medicines, and better living, returned to the state he was in just a year before, remaining perfectly stationary.

By one of those fortuitous accidents which occasionally occur in the mind of every medical man, when vaccilating between first one remedial measure and then another, scarcely knowing which satisfactorily to adopt; recalling also to mind the motto of a worthy friend, *Dum spiro spero*, I determined to examine the patient again. As to the pain he had originally suffered in his back, he strongly assured me, that until his journey in the cart, he had felt no pain or even uneasiness there; but that since, he had occasionally an obscure aching, and pointed to the lumbar vertebræ as well as he could. On surveying the spot, not the least deviation from healthy form was perceptible, no aggravation of pain by pressure took place; however, I determined on applying a caustic on each side of the pained part. On June 10, this was done, and the eschars falling out about the usual time, each cavity admitted four peas; they were daily embued with ung. ærugin. the sores discharged freely; all medicine whatever was most gladly laid aside, and of the other external means, friction only continued. The situation of the issues made it necessary to touch them weekly with caustic. In about three months he became able to turn in bed without assistance, and lie on either side, which he had not done for so many years before; appetite, strength, spirits and freedom of motion, so far returned, that at the end of six months, the arms and left leg were become perfectly well; he could then go tolerable easy and quick on his crutches. A few days since, he walked to my house with an under hand stick only, and is now in all respects so nearly recovered, as to make it a matter of indifference whether he uses any support or not; the sores are kept discharging as at first.

It will doubtless be readily observed, that Edwards's is a solitary case of the good effects of topical stimuli in the form of caustic, in a case bearing but slight resemblance to those for which this noble remedy has now so long and so successfully been employed.—It is so.—I have none other at present to produce, in aid of the practice; but I cannot help thinking, and I hope without presumption, that when all its circumstances are considered, it was better to publish it, under the wish and belief, that by so doing the number will soon be increased. If this argument be not sufficient, in justification for occupying the time of the Reader, and the pages of the Journal, I can only say, the very common and distressing nature of the disease in question, with the little relief afforded by medicine, must stand forward as my apology.

I should have before observed, that this cripple was electrified at one of the Infirmaries, but not regularly; he was likewise galvanized, under my direction, three or four times from a powerful apparatus, skilfully applied by Mr. Dalton of Liverpool, when delivering a course of lectures here last spring; but complaining of being *dreadfully burnt*, at the part where the tin foil was applied, and feeling so oddly all over him, I could not prevail upon him to continue the process, although he acknowledged a belief that it would have done him good—"could he have stood the burning." The great length of time this affection subsisted, seems to prove decisively, that the mischief, if any, was formed in the vertebræ, was not precisely analagous to that description of case so accurately delineated by Mr. Pott, or its ravages must have been more apparent, and its symptoms more correspondent to the osseous malady; the flexor muscles of the lower limbs acted with such a force, that no exertion could straiten them in the least, without insupportable agony.—Mr. Pott tells us, he never saw a case of Palsy of the lower limbs in a subject above 40; he also says, the spinal curvature is "always from within outwards." Had Edwards a curvature, it must have been the reverse, and external proportionate depression would have been more or less manifest, which was by no means the case; the useless state of the limbs is not necessarily preceded by any evident alteration in the figure of the vertebræ, but the result of disease attacking the part; which disease, when removed, the limbs become restored, whether the spinal deformity be, or be not, removed.

I am, &c.

*Chester, March* 10, 1806. GEO. N. HILL.

*P. S.* Your Number for December last, informs the public, "that any paper in answer to the first question for the the Fothergilian Medal for the year ensuing, will be received at the Society's House, Bolt Court, Fleet Street, any time previous to the 31st day of January, 1806."—But I was not able to find in any preceding Number, a statement of the subject of the question to be written upon; was this an oversight by the person who conveyed the intelligence?

